# Perceived Coach-Created Motivational Climates as Predictors of Athletes’ Goal Reengagement: The Mediational Role of Goal Motives

**DOI:** 10.3389/fpsyg.2021.740060

**Published:** 2021-12-24

**Authors:** Natalia Martínez-González, Francisco L. Atienza, Inés Tomás, Isabel Balaguer

**Affiliations:** ^1^Department of Social Psychology, Faculty of Psychology, University of Valencia, Valencia, Spain; ^2^Department of Personality, Evaluation and Psychological Treatment, Faculty of Psychology, University of Valencia, Valencia, Spain; ^3^Department of Methodology of Behavioral Sciences, Faculty of Psychology, University of Valencia, Valencia, Spain

**Keywords:** motivational climate, goals, motives, reengagement, athletes, empowering climate, disempowering climate

## Abstract

Athletes have to face several challenges during the sport season, and one of them could involve dealing with unattainable goals. In these situations, being able to reengage in other goals as a form of goal adjustment and in response to contextual demands is adaptive. According to previous literature, some aspects of the athletes’ social context, such as coach-created motivational climates, could encourage more adaptive responses in athletes, and so it is possible that these climates would also promote athletes’ goal regulation and goal reengagement. The purpose of this study was twofold: to analyze whether athletes’ perception of empowering and disempowering climates were related to their goal reengagement through the mediation of goal motives; and to examine the interaction between the two climates when they predict reengagement through athletes’ goal motives. Participants were 414 Spanish university athletes (49.5% male, 50.5% female) who belonged to different university teams, with ages ranging from 17 to 33 years old (*M* = 20.61, *SD* = 2.58). In the sport facilities, all of them completed questionnaires that evaluated their perception of empowering and disempowering climates, their goal motives, and their goal reengagement. Structural equation modeling (SEM) results showed that perceived empowering climate positively predicted autonomous goal motives, which in turn had a positive relationship with goal reengagement. Conversely, perceived disempowering climate positively predicted controlled goal motives, which were not related to goal reengagement. Thus, we only found support for the indirect relationship between perceived empowering climate and goal reengagement through autonomous goal motives. Moderated mediation analyses revealed that interaction effects between perceived empowering and disempowering climates were not significant in the prediction of goal reengagement through goal motives. Findings revealed that the perception of empowering climates promotes athletes’ goal reengagement when goals become unattainable via the increase in their autonomous goal motives. Conversely, when athletes perceive disempowering climates, they have more controlled goal motives, which are not related to goal reengagement. In addition, the study supports the need to educate coaches to create more empowering and less disempowering climates.

## Introduction

Across the lifespan, people are continually pursuing personal goals in different contexts, which is a way for them to organize their behavior and even mature as individuals (Sheldon, 2014). However, in the process of pursuing valuable goals, people may encounter obstacles or changes in the context that make their goal attainment more difficult, sometimes threatening their well-being. In these situations, some people adapt and maintain their psychological health and well-being better than others, even when their important goals are frustrated (Barlow et al., 2020). In sport, athletes have to deal with the challenges of the competitive context, where personal goals may sometimes not be feasible due to injuries, biological capabilities, or time constraints (Ntoumanis et al., 2014a). Understanding the functioning of effective self-regulation processes when goals are unattainable in sport, and how coaches can enhance athletes’ responses, could be the key to promoting athletes’ well-being and maintaining good functioning during the season.

Although some past literature in different contexts has defended the importance of perseverance in personal goals and confidence in their attainability (e.g., Emmons, 1986; Taylor and Brown, 1988), more recent evidence has shown that in situations where goals become unattainable, it is more adaptive to give them up and start a process to search for and engage in other goals (Wrosch et al., 2003a; Wrosch, 2011). In fact, evidence suggests that when people are highly committed to unattainable goals, the positive impact of goal commitment on well-being disappears or even becomes negative (Boudrenghien et al., 2012) because they invest personal resources without getting results and, thus, accumulate failure experiences. If some people in these situations are not able to achieve goal adjustment, they are likely to experience a decrease in their quality of life (Wrosch et al., 2013), an increase in psychological distress levels (Carver and Scheier, 1999; Wrosch et al., 2007a), or even depressive symptoms (Brandtstädter and Renner, 1990). Two distinct self-regulation capacities are involved in goal adjustment: goal disengagement and goal reengagement. Whereas goal disengagement implies abandoning the unattainable goals, goal reengagement refers to the capacity to identify, commit to, and pursue alternative goals when current goals become unattainable (Wrosch et al., 2003b).

Research with different populations (e.g., general, clinical, school) has largely demonstrated that both capacities, by serving distinct mechanisms, contribute to a person’s quality of life (Wrosch, 2011), predicting effective biological functioning, subjective well-being, and mental and physical health (e.g., Wrosch et al., 2003b,2007b; Brassen et al., 2012). More specifically, goal disengagement, which implies investing less time and effort in unattainable goals, is adaptive because it involves less emotional distress and fewer health problems (Mens et al., 2015; Jobin and Wrosch, 2016). These findings suggest that disengagement protects individuals from negative psychological states associated with experiences of failure (Wrosch et al., 2007b), whereas goal reengagement has been related to both low levels of negative indicators and high positive levels of subjective well-being (North et al., 2014; Haase et al., 2021). These possible benefits of goal reengagement stem from the fact that pursuing meaningful alternative goals fosters people’s purpose in life, positive affect, and life satisfaction (Barlow et al., 2020), and it promotes a sense of coherence and feelings of control (Ryff and Keyes, 1995). Although there is less research than in other contexts, the investigation in the sport domain has found that, in line with findings in other domains, goal reengagement (and not disengagement) predicts greater psychological well-being in athletes (Nicholls et al., 2016). Therefore, and given that sport is a highly goal-driven environment, more explorations about goal adjustment, in terms of goal reengagement and disengagement, in this context are necessary (Healy et al., 2018).

Various studies over the years have expressed the need to identify the conditions that facilitate these processes of goal adjustment (e.g., Wrosch et al., 2013). However, until now, researchers have focused more on studying and identifying predictors of goal disengagement (e.g., Wrosch and Miller, 2009; Koppe and Rothermund, 2017), whereas less is known about goal reengagement and its predictors (Wrosch and Scheier, 2020). Based on this background, recent research projects have pointed out the need to study which variables can predict individual differences in the capacity to reengage in new goals (e.g., Haase et al., 2021), and how reengagement capacities can be developed over time through suitable training (Herrmann et al., 2019).

Previous literature on sport has suggested that goal motives could be involved in the process of athletes’ goal reengagement (Ntoumanis et al., 2014b; Smith and Ntoumanis, 2014; Smith, 2016). According to the self-concordance model (SCM; Sheldon and Elliot, 1999), depending on whether people’s goals are more or less concordant with their interests and values, the motives that underlie goal striving (called goal motives) can be differentiated as autonomous or controlled. Autonomous goal motives are aligned with an individual’s personal values, being perceived as important and enjoyable. In contrast, controlled goal motives are regulated by pressures from external (others’ expectations) or internal (guilt) factors. Evidence has revealed that when athletes pursue goals for autonomous motives, they have greater psychological and physical well-being (Smith et al., 2011; Healy et al., 2014). Conversely, controlled goal motives have been positively related to subjective ill-being (Healy et al., 2014; Gaudreau and Braaten, 2016), whereas other results have shown that they are unrelated or negatively related to athletes’ well-being (Smith et al., 2007, 2010; Healy et al., 2014). Some literature has highlighted the importance of coaches and their influence on athletes’ goal pursuits with more autonomous or controlled motives. In this line, Smith et al. (2007) found that when coaches supported athletes’ autonomy, athletes reported more autonomous goal motives and need satisfaction, which in turn led to greater psychological well-being. These findings support the self-determination theory (SDT; Deci and Ryan, 1985, 2000) proposal about how a person in a position of authority (e.g., a coach), through autonomy support, can provide opportunities for choice and volition to others (e.g., athletes) while minimizing pressure (Black and Deci, 2000). Previous literature within this framework showed that the social environment can contribute to the use of the appropriate self-regulatory strategy, given that it is able to help people to openly experience events and reflectively and congruently choose and regulate behavior (Ryan and Deci, 2017). In fact, it has been reported that autonomy-supportive climates allow individuals to self-regulate in the process of goal pursuit, facilitating goal adjustment or even resetting goals (Koestner et al., 2015). In addition, feelings of competence would lead to athletes’ belief that they can meet the demands of the situation (Blascovich, 2008). Therefore, coaches, through their behaviors, could influence their athletes’ strategies when a goal becomes unattainable and they have to adapt. Smith and Ntoumanis (2014) studied the relationships between coach behaviors and athletes’ responses to unattainable goals through athletes’ goal motives. They found that perceptions of autonomy-support positively predicted the intention to reengage in alternative goals through autonomous goal motives. In contrast, athletes’ perceptions of controlling coach behaviors did not positively or negatively predict goal reengagement. Although more research is needed, the existing evidence suggests that coaches can play an important role in promoting athletes’ self-regulation by assisting athletes in pursuing goals regulated by autonomous motives and fostering their goal reengagement (Kitsantas et al., 2018).

In addition to the autonomy support and controlling behaviors mentioned above, other dimensions of the motivational climate created by the coach might help us to obtain complementary information about the promotion of goal motives and goal reengagement. Specifically, it would be interesting to know whether the concept of competence promoted by the coach contributes to this question. Achievement Goal Theory has established (AGT; Nicholls, 1989; Ames, 1992) that the climate the coaches create promotes more adaptive or maladaptive emotions, thoughts, and behaviors (Duda and Balaguer, 2007). On the one hand, task-involving climates promote task mastery and cooperation, emphasize effort (Nicholls, 1989), and lead athletes to more self-determined motivation (e.g., Balaguer et al., 2011). Thus, when athletes are in task-involving climates, their perceptions of competence are based on self-referenced criteria, which are more within the individual’s control (Duda, 2001) and could, therefore, also lead to more autonomous goal motives. On the other hand, ego-involving climates increase rivalry and cause athletes to focus on normative comparison rather than on the process, promoting maladaptive outcomes and reducing self-determined motivation (Álvarez et al., 2009; López-Walle et al., 2011). These normative-based criteria to judge competence lead athletes to focus on outcomes that are outside their personal control, such as attaining social approval or external rewards (Reinboth and Duda, 2006); therefore, they could enhance controlled goal motives.

A growing body of conceptual and empirical research in sport psychology addressing the coach-created motivational climate dimensions of SDT (autonomy support and controlling) and AGT (task-involving climate and ego-involving climate) developed by Duda (2013) can be helpful to continue to clarify the relationship between other dimensions of the motivational climate created by the coach with goal motives and goal reengagement. Based on the theoretical principles of these theories, Duda’s model proposes a hierarchical and multidimensional conceptualization of the coach-created motivational climate, suggesting that it could be more or less empowering and more or less disempowering. In addition to the dimensions discussed above, Duda also introduces social support as a positive dimension that contributes to athletes’ optimal functioning. Specifically, the author argues that when coaches bring social support to their athletes, they are willing to help them and give them confidence (Duda et al., 2018), and this will have positive consequences for them (Sheridan et al., 2014). Integrating all these dimensions, an empowering climate is characterized by high task-involving, autonomy supportive, and social supportive behaviors, whereas a disempowering climate includes high degrees of ego-involving and controlling behaviors (Duda, 2013; Duda and Appleton, 2016). Duda and her colleagues (Duda, 2013; Duda et al., 2018), in their proposed model of features of empowering and disempowering motivational climates, stated that empowering climate is an antecedent of autonomous motivation, whereas disempowering climate is an antecedent of controlled motivation. Thus, this model offers the rational to support the idea that these climate dimensions could be related to autonomous or controlled goal motives and optimal or compromised functioning.

Existing literature based on this model shows that perceived empowering climates are related to adaptive outcomes, whereas perceived disempowering climates are related to maladaptive ones (e.g., Smith et al., 2015, 2017; Appleton and Duda, 2016; Krommidas et al., 2016; Zourbanos et al., 2016; Castillo et al., 2017; Fenton et al., 2017; Mosqueda et al., 2019). Two of these studies (Fenton et al., 2017; Mosqueda et al., 2019) found that an empowering climate positively predicts sport-related enjoyment through the mediation of autonomous motivation. In this line, another study (Castillo et al., 2017) reported that perceived empowering climate promotes athletes’ satisfaction of basic psychological needs of competence, autonomy, and relatedness, and this in turn enhances their self-determined motivation. Taking these two climate dimensions into account, the study conducted by Zourbanos et al. (2016) examined the relationship between perceived empowering and disempowering climates and athletes’ self-efficacy. They found that only perceived empowering climate was a positive predictor, whereas no relationship emerged between this variable and a disempowering climate. Furthermore, perceived empowering climate was a positive predictor of self-reported health, life satisfaction, subjective vitality, and enjoyment, whereas perceived disempowering climate negatively predicted life satisfaction and enjoyment (Krommidas et al., 2016). Finally, with regard to athletes’ motivation and emotional experiences, Ruiz et al. (2021) recently found that the perception of an empowering climate predicted athletes’ autonomous motivation and pleasant emotions (i.e., happiness, excitement), whereas perceived disempowering climate predicted controlled motivation and unpleasant emotions (i.e., anxiety, dejection, anger).

In order to extend the previous literature on the relationship between the social environment and the use of the appropriate self-regulatory strategy, in the current study, the empowering and disempowering dimensions of motivational climate are used. Based on the conceptual model by Duda et al. (2018) and literature mentioned above that supports the relationship between perceived empowering climate and adaptive outcomes and between disempowering climate and less adaptive ones (e.g., Krommidas et al., 2016; Fenton et al., 2017), we propose that athletes who perceive a more empowering climate would be more capable of (re) engaging in new or alternative goals. By contrast, the perception of a disempowering climate would not encourage this self-regulatory capacity. Furthermore, taking into account reported evidence that coaches’ autonomy supportive behavior is related to athletes’ autonomous goal motives, whereas the prevalence of coaches’ controlling behaviors is related to a greater probability that athletes will present controlled goal motives (Smith et al., 2010; Healy et al., 2014; Smith and Ntoumanis, 2014), in the current research, these relationships will be tested along with empowering and disempowering climates. It is important to highlight that empowering and disempowering climates can co-exist, and that a coach-created climate can to some degree be both (Tessier et al., 2013; Smith et al., 2015, 2017). Based on this premise, some researchers have examined the interaction between these climate dimensions, finding, for example, that an empowering climate significantly moderates the debilitating effects of a disempowering climate on outcomes such as enjoyment, reduced accomplishment, and physical symptoms (Appleton and Duda, 2016).

In sum, the purpose of this study is to analyze whether athletes’ perceptions of empowering and/or disempowering motivational climates predict athletes’ reengagement, and the role of autonomous and controlled goal motives as mediators. Specifically, we will examine the pathway through which the perceived coach-created motivational climate could be related to athletes’ individual responses of goal reengagement when faced with unattainable goals through the mediation of their goal motives. Additionally, the interaction between the two climates in predicting athletes’ autonomous and controlled goal motives will also be explored.

Specifically, the objectives and hypotheses of this study are the following:

Objective 1: Analyze the predictive role of perceived empowering and disempowering climates in goal reengagement through the meditation of athletes’ goal motives (autonomous and controlled).

Objective 2: Explore the interaction between perceived empowering and disempowering climates when climates predict reengagement through athletes’ goal motives (autonomous and controlled).

Hypothesis 1: Athletes’ perceptions of motivational climates will predict goal motives, which in turn will predict reengagement.
H1a. Athletes’ perceptions of empowering climate will positively predict autonomous goal motives, which in turn will positively predict goal reengagement. Conversely, perceived empowering climate will negatively predict controlled goal motives, which in turn will negatively predict goal reengagement.
H1b. Athletes’ perceptions of disempowering climate will negatively predict autonomous goal motives, which in turn will positively predict goal reengagement. Conversely, perceived disempowering climate will positively predict controlled goal motives, which in turn will negatively predict goal reengagement.
Hypothesis 2: Perceived empowering and disempowering climates will interact to predict goal reengagement through goal motives (autonomous and controlled).
H2a. Perceived disempowering climate will diminish the positive relationship between perceived empowering climate and goal reengagement through autonomous and controlled goal motives.
H2b. Perceived empowering climate will buffer the negative relationship between perceived disempowering climate and goal reengagement through autonomous and controlled goal motives.

## Materials and Methods

### Participants

The participants were 414 athletes (49.5% male, 50.5% female) from different university teams that compete in the Regional University Sports Championship (CADU), which takes place annually in the Valencian Community (Spain). The age range of the athletes was between 17 and 33 years (*M* = 20.61, *SD* = 2.58). A total of three Valencian universities, with twelve sports teams each, participated in the research, including the male and female basketball, handball, football, indoor football, rugby, and volleyball teams. All athletes were recruited by the sport services at the beginning of the sport season to form part of the college team, and at the time of data collection, they had had at least 4 weeks of interaction with their coach an average of 1.8 ± 0.78 h per week.

### Procedure

Before the data collection, the research obtained ethical approval from the university Human Research Ethics Committee (Procedure number: 1129330). Afterward, researchers requested the participation of each university sport service and programmed the instrument administration during regular training sessions. Because of the specific demands of the Spanish language, the questionnaires were adapted to refer to male or female using the corresponding conjugations in each case.

During the month of November 2019, trained researchers collected data in the sports facilities of each university (at the training field or in rooms equipped with tables and chairs). Prior to questionnaire administration, athletes were informed about the procedure, which guaranteed confidentiality and anonymity, and they signed a consent form to participate voluntarily. The time spent on completion was approximately 25 min.

### Instruments

Athletes’ perceptions of empowering and disempowering climate were measured using the Spanish version^1^ of the Coach-created Empowering and Disempowering Motivational Climate Questionnaire (EDMCQ-C; Appleton et al., 2016). The questionnaire contains 34 items, 17 corresponding to Empowering Climate and 17 corresponding to Disempowering Climate. On the one hand, the empowering climate scale is composed of three subscales: task-involving climate (9 items; “My coach encouraged players to try new skills”), autonomy-supportive coach (5 items; “My coach gave players choices and options”), and socially supportive coach (3 items; “My coach could really be counted on to care, no matter what happened”). On the other hand, the disempowering climate scale is composed of two subscales: ego-involving (7 items; “My coach substituted players when they made a mistake”) and controlling coaching (10 items; “My coach was less friendly with players if they didn’t make the effort to see things his/her way”). Participants rated the extent to which these behaviors had been present on this team in the past 3–4 weeks, using a 5-point Likert scale from 1 (*strongly disagree*) to 5 (*strongly agree*). Both the original and Spanish versions have shown adequate validity and reliability (α = 0.82 to 0.90) in previous studies with athletes (e.g., Appleton and Duda, 2016; Appleton et al., 2016; see text footnote 1; Castillo et al., 2017).

The Spanish version of the Goal Motives Questionnaire (Martínez-González et al., 2021a) was used to assess the athletes’ personal goal motives. Based on the idiographic methodology proposed by Sheldon (2002), and following the procedures used in previous studies with athletes (e.g., Smith et al., 2007, 2011; Smith and Ntoumanis, 2014), this questionnaire was adapted to measure personal goals in the Spanish sport domain. Although it was created recently, the first data have shown acceptable validity and reliability (α = 0.67 and 0.70) (Martínez-González et al., 2021a). Athletes were instructed to “identify your most important sporting goal that you hope to make progress on during the current season,” and then they had to rate the extent to which they were striving for the goal for external (two items; e.g., “Because someone else wants me to”), introjected (two items; e.g., “Because I would feel ashamed, guilty, or anxious if I didn’t”), identified (two items; e.g., “Because I personally believe it’s an important goal to have”), and intrinsic (two items; e.g., “Because of the fun and enjoyment the goal provides me”) motives. All responses were rated on a 7-point Likert scale from 1 (*Not at all*) to 7 (*Very much so*). Consistent with past research (e.g., Healy et al., 2014; Ntoumanis et al., 2014b), controlled and autonomous goal motives were calculated by aggregating the introjected and external scores and the intrinsic and identified scores, respectively.

Athletes’ goal reengagement was measured with six items corresponding to the reengagement subscale from the Spanish version (Soubrier et al., 2017) of the Goal Adjustment Scale (GAS; Wrosch et al., 2003b). Past literature using this subscale found adequate validity and internal consistency, with alphas ranging between 0.87 and 0.94 in Spanish samples (Soubrier et al., 2017; Ramírez-Maestre et al., 2019). Regarding the six items that assess the capacity to reengage in new goals, two items focus on the intention to identify new goals (e.g., “I think about other new goals to pursue”), two on the intention to commit to new goals (e.g., “I tell myself that I have a number of other new goals to draw on”), and two on intentions to begin actively pursuing new goals (e.g., “I start working on other new goals”). Corresponding to the aims of this research, the generic stem used put the respondents in the situation of having to stop pursuing an important goal because it is unattainable. Participants responded on a Likert scale ranging from 1 (*never*) to 5 (*always*). In the current study, the item “I convince myself that I have other meaningful goals to pursue” was removed, following the recommendations of the authors of the Spanish version (i.e., Soubrier et al., 2017).

### Data Analysis

**FIGURE 1:**
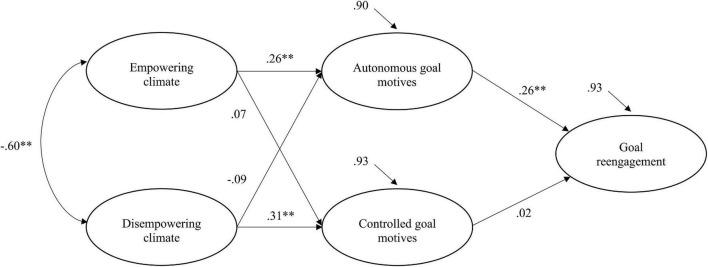
Structural equation model of the associations between empowering and disempowering climates, goal motives, and goal reengagement. Statistics are standardized regression coefficients, ^**^p < 0.01.

First, preliminary analyses, such as the estimation of descriptive statistics, scale reliability coefficients, and bivariate correlations among the variables of interest, were carried out using the IBM SPSS Statistics 25 software. Second, to test Hypothesis 1, structural equation modeling (SEM) with latent variables was performed using Mplus (Version 7; Muthén and Muthén, 2012) to check a model that included all the relationships hypothesized in H1a and H1b (see Figure 1). Specifically, maximum likelihood was used as the estimation method, provided there was a normal distribution of the variables (skewness and kurtosis values in the range +1/−1). Model fit was assessed using the following indices: chi-square (χ^2^), the Comparative Fit Index (CFI), the Tucker-Lewis Index (TLI), the Standardized Root Mean Square Residual (SRMR), and the Root Mean Square Error of Approximation (RMSEA). The cut-off points used to indicate an acceptable fit were: CFI and TLI > 0.90, and SRMR and RMSEA < 0.08 (Hu and Bentler, 1999). In addition, the significance of indirect effects was tested by using bias corrected (BC) bootstrap 95% confidence intervals (CI), as implemented in Mplus. If the confidence intervals did not include zero, mediation was supported.

**FIGURE 2:**
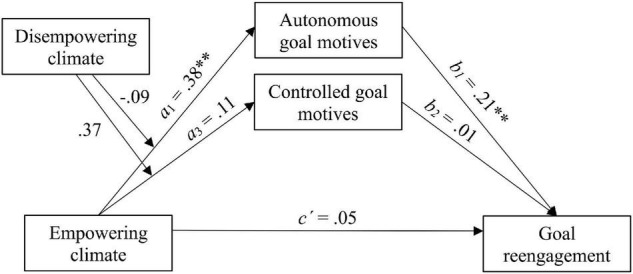
Moderated mediation model of the effect of empowering climate on goal reengagement through goal motives, ^**^p < 0.01.

**FIGURE 3:**
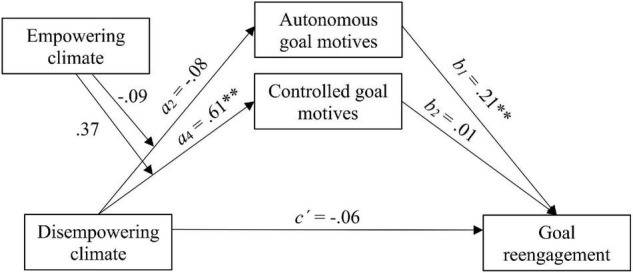
Moderated mediation model of the effect of disempowering climate on goal reengagement through goal motives, ^**^p < 0.01.

Finally, to test Hypothesis 2, two moderated mediation models were carried out using the PROCESS macro (model 7) for SPSS version 3.5 (Hayes, 2018). We used observed variables to test the second hypothesis due to simplicity. Prior to the analysis, the means of variables that defined the products were centered. Figures 2, 3 represent the moderated mediation models hypothesized in H2a and H2b, respectively. The total, direct, indirect, and conditional indirect effects of empowering and disempowering climates on goal reengagement were analyzed. Specifically, the bootstrapping method based on 5,000 samples was used to assess the significance of indirect and conditional indirect effects.

## Results

### Preliminary Analysis

All the participants provided complete data for the variables of interest. Analysis of outliers was carried out through Z-scores, and the criterion applied was to consider values higher than ± 3.29 extreme values (Field, 2013; Tabachnick and Fidell, 2013). This procedure identified six participants who showed extreme values for: goal reengagement (3 participants), empowering climate (2 participants), and autonomous goal motives (1 participant). After removing these participants, using the univariate trimming method, the final sample consisted of 408 athletes.

### Descriptive Statistics, Reliabilities, and Bivariate Correlations

An inspection of skewness and kurtosis shows that most of the coefficients were in the range of (−1, 1) recommended for normal-distributions (Muthén and Kaplan, 1992; Ferrando and Anguiano-Carrasco, 2010). The values for autonomous goal motives were an exception because they were a little higher but within the acceptable range of (−1.5, 1.5) (Forero et al., 2009). Mean scores revealed that athletes perceived high empowering climate and low disempowering climate. Moreover, they reported high levels of autonomous goal motives and low levels of controlled goal motives. Finally, athletes’ goal reengagement showed medium levels (see Table 1).

**TABLE 1:** Descriptive statistics, reliabilities, and bivariate correlations.

	Range	*M*	*SD*	α	Skewness	Kurtosis	1	2	3	4	5
Empowering climate	1–5	4.10	0.48	0.90	−0.32	−0.01	–				
Disempowering climate	1–5	2.37	0.56	0.84	−0.01	−0.46	−0.50**	–			
Autonomous goal motives	1–7	6.32	0.75	0.70	−1.30	1.34	0.27**	−0.18**	–		
Controlled goal motives	1–7	2.47	1.20	0.71	0.56	−0.46	−0.09	0.26**	−0.19**	–	
Goal reengagement	1–5	3.58	0.72	0.83	−0.20	−0.10	0.09	0.08	0.24**	−0.05	–

**p < 0.01.

Cronbach’s alpha coefficients (see Table 1) revealed satisfactory reliability for the empowering and disempowering climate, controlled goal motives, and goal reengagement scales. In the case of autonomous goal motives, the item “Because it teaches me self-discipline” was removed, and so the reliability coefficient increased (from 0.63 to 0.70), which was acceptable.

With regard to the relationships among the study variables, bivariate correlations indicated that empowering climate correlated significantly and positively with autonomous goal motives, whereas the relationship with controlled goal motives was not significant. Conversely, disempowering climate was significantly related to both goal motives, with this relationship being negative with autonomous motives and positive with controlled motives. Moreover, the relationship between empowering and disempowering climate was significant and negative, consistent with the model framework (Appleton and Duda, 2016). Regarding goal reengagement, only its positive correlation with autonomous goal motives was significant (see Table 1).

### Structural Equation Modeling

The hypothesized model that comprises the associations between empowering and disempowering climate, goal motives, and goal reengagement was tested with SEM (see Figure 1). Empowering and disempowering climate items were parceled in order to maintain an acceptable ratio of number of participants per estimated parameter (Bentler and Chou, 1987). Specifically, the 17 empowering climate items were parceled in three indicators, forming one indicator for each subscale (task-involving climate, autonomy-supportive coach, and socially supportive coach), whereas the 17 disempowering climate items were parceled in two indicators that corresponded to their subscales (ego-involving climate and controlling coach).

Results indicated that the model had an acceptable fit to the data: χ^2^ (112) = 293.59, *p* < 0.05, RMSEA = 0.06, CFI = 0.93, TLI = 0.91, SRMR = 0.06. Empowering climate positively predicted autonomous goal motives (*a*_1_ = 0.26, *p* < 0.01), which in turn positively predicted goal reengagement (*b*_1_ = 0.26, *p* < 0.01). Conversely, disempowering climate positively predicted controlled goal motives (*a*_4_ = 0.31, *p* < 0.01), which not was significantly related to goal reengagement (*b*_2_ = 0.02, *p* > 0.05). Empowering climate did not have a significant relationship with controlled goal motives (*a*_3_ = 0.07, *p* > 0.05), and disempowering climate did not have a significant relationship with autonomous goal motives (*a*_2_ = −0.09, *p* > 0.05). Moreover, empowering and disempowering climates were negatively related (β = −0.60, *p* < 0.01), consistent with previous research (Appleton and Duda, 2016). Regarding the effect sizes, R-squares were 0.10 (*p* < 0.01) for autonomous goal motives, 0.07 for controlled goal motives (*p* < 0.05), and 0.07 for goal reengagement (*p* < 0.05).

Results of bias corrected (BC) bootstrap 95% confidence intervals (CI) supported the indirect effect of empowering climate on goal reengagement through autonomous goal motives (IE_a1b1_ = 0.10; BC bootstrap 95% CI = [0.02, 0.20]), but not through controlled goal motives (IE_a3b2_ = 0.002; BC bootstrap 95% CI = [−0.02, 0.02]). Furthermore, the indirect effect of disempowering climate on goal reengagement was not significant through autonomous goal motives (IE_a2b1_ = −0.002; BC bootstrap 95% CI = [−0.09, 0.04]) or through controlled motives (IE_a4b2_ = 0.005; BC bootstrap 95% CI = [−0.04, 0.05]).

### Moderated Mediation Model

Results of moderation analyses for the two tested models showed that the interactions between empowering and disempowering climate were not significant in the prediction of autonomous goal motives (Interaction = −0.09, *p* > 0.05) or controlled goal motives (Interaction = 0.37, *p* > 0.05). The conditional indirect effects (CIE) were not statistically significant, as indicated by the moderated mediation indices for both autonomous goal motives (CIE = −0.020; bootstrap 95% CI = [−0.07, 0.04]) and controlled goal motives (CIE2 = 0.002; bootstrap 95% CI = [−0.02, 0.03]). Accordingly, the conditional indirect effect at different values of the moderator did not change in any of the models (see Tables 2, 3). These results indicated that perceived disempowering climate did not moderate the relationship between perceived empowering climate and goal reengagement through autonomous and controlled goal motives. In the same way, perceived empowering climate did not moderate the negative relationship between perceived disempowering climate and goal reengagement through autonomous and controlled goal motives.

**TABLE 2:** Conditional indirect effects of moderated mediation model of the effect of empowering climate on goal reengagement through goal motives.

Indirect effect	Disempowering (−1SD, Mean, +1SD)	Indirect effect (SE)	LL 95% CI	UL 95% CI
Empowering climate → Autonomous goal motives → Goal reengagement	−0.56 0.00 0.56	0.09 (0.03) 0.08 (0.02) 0.07 (0.03)	0.04 0.04 0.02	0.15 0.13 0.13

Empowering climate → Controlled goal motives → Goal reengagement	−0.56 0.00 0.56	−0.00 (0.01) 0.00 (0.01) 0.00 (0.01)	−0.01 −0.01 −0.02	0.01 0.01 0.02

*SE = standard error. LL95%CI = lower limit of 95% confidence interval; UL95%CI = upper limit of 95% confidence interval.*

**TABLE 3:** Conditional indirect effects of moderated mediation model of the effect of disempowering climate on goal reengagement through goal motives.

Indirect effect	Empowering (−1SD, Mean, +1SD)	Indirect effect (SE)	LL 95% CI	UL 95% CI
Disempowering climate → Autonomous goal motives → Goal reengagement	−0.48 0.00 0.48	−0.01 (0.02) −0.02 (0.02) −0.03 (0.02)	−0.05 −0.05 −0.06	0.04 0.01 0.01

Disempowering climate → Controlled goal motives → Goal reengagement	−0.48 0.00 0.48	0.00 (0.01) 0.00 (0.02) 0.01 (0.02)	−0.02 −0.03 −0.04	0.03 0.04 0.05

*SE = standard error. LL95%CI = lower limit of 95% confidence interval; UL95%CI = upper limit of 95% confidence interval.*

With regard to mediation, consistent with previous results, the findings confirmed that empowering climate positively predicted autonomous goal motives (*a*_1_ = 0.38, *p* < 0.01), but it did not significantly predict controlled goal motives (*a*_3_ = 0.11, *p* > 0.05). Regarding goal reengagement, it was positively predicted by autonomous goal motives (*b*_1_ = 0.21, *p* < 0.01), but not by controlled goal motives (*b*_2_ = 0.01, *p* > 0.05). Moreover, the direct effect of empowering climate on goal reengagement was not significant (*c′* = 0.05, *p* > 0.05), whereas the indirect effect through autonomous goal motives (but not controlled) was significant (see Figure 2 and Table 2). Moreover, results with disempowering climate as a predictor showed that disempowering climate positively predicted controlled goal motives (*a*_4_ = 0.61, *p* < 0.01), but this in turn did not significantly predict goal reengagement (*b*_2_ = 0.01, *p* > 0.05). In addition, disempowering climate did not predict autonomous goal motives (*a*_2_ = −0.08, *p* > 0.05), but autonomous goal motives significantly predicted goal reengagement (*b*_1_ = 0.21, *p* < 0.01). Neither the interaction between the climates nor the direct effect of disempowering climate on goal reengagement (*c′* = −0.06, *p* > 0.05) was significant. Neither of the indirect effects were significant (see Figure 3 and Table 3).

## Discussion

Literature has largely demonstrated that a self-regulation process that has been related to a wide range of adaptive outcomes in situations where important goals become unattainable is goal reengagement (Wrosch et al., 2003b). In sport contexts, being able to carry out this type of self-regulation is essential because athletes are continually driven by goals (Healy et al., 2018), and some of them become unattainable. In these situations, goal reengagement is an alternative. Previous evidence has shown that goal reengagement can be developed over time and with repeated experience with unattainable goals (Wrosch and Miller, 2009; Mens et al., 2015), which may be due in part to internal (Haase et al., 2021) or external factors (Wrosch et al., 2003b; Smith and Ntoumanis, 2014). The external factors, and specifically the role of coach-created motivational climates and their relationship with athletes’ goal motives and goal reengagement, are the principal interest of the present study. Taking into account that previous studies reported that athletes’ perceptions of coach autonomy-support positively predicted the intention to reengage in alternative goals through autonomous goal motives, whereas perceptions of controlling coach behaviors did not positively or negatively predict goal reengagement, in this paper these and other dimensions of motivational climate are incorporated in order to have a broader vision of the phenomenon under study. Based on Duda’s 2013 and Duda et al. (2018) model of empowering and disempowering motivational climates, the main interest of the current study was to analyze whether athletes’ perceptions of empowering and disempowering climates could predict their goal reengagement through the mediation of their goal motives (Objective 1). Furthermore, we examined whether empowering and disempowering climates interacted in predicting goal reengagement through goal motives (Objective 2).

Regarding the first hypothesis formulated, a model was tested that examined the relationships between perceived empowering and disempowering climates, goal motives, and goal reengagement. Results showed that the perception of an empowering climate predicted athletes’ autonomous goal motives, which in turn predicted their goal reengagement. Conversely, the perception of a disempowering climate predicted athletes’ controlled goal motives, which were not related to athletes’ goal reengagement. These findings partially support the sub-hypotheses and add evidence about goal reengagement and how it can be promoted by coaches who enhance their athletes’ autonomous goal motives, as previous researchers have suggested, creating adaptive motivational climates (e.g., Smith and Ntoumanis, 2014). In the current study, additional dimensions of motivational climate included in the higher dimension of empowering climates are explored. Therefore, we can state that not only coaches’ autonomy support, but also coaches’ promotion of task-involving climates and social support, lead athletes to have more autonomous goal motives. These findings imply that, when coaches carry out autonomy supportive behaviors, they enhance self-referenced criteria to judge athletes’ competence, creating task-involving climates, and when they provide athletes with an environment of confidence through social support, athletes are more likely to engage in goals with autonomous motives. That is, when athletes perceive empowering climates, they pursue goals that are more concordant with their personal values and interests. In contrast, the perception of a disempowering climate leads athletes to pursue goals that are more regulated by external or internal pressures. The latter means that coaches who have coercive, pressuring, and autocratic behaviors typical of a controlling interpersonal style lead athletes to more controlled goal motives, as previous literature has shown (Smith and Ntoumanis, 2014), and this also occurs when coaches create ego-involving climates. This suggests that, as other studies have revealed, a focus on outcomes located outside of the athletes’ personal control is fostered when coaches promote rivalry among athletes, comparison, and normative-based criteria to assess competence (Reinboth and Duda, 2006), and this also seems to be the case with controlled goal motives.

Regarding goal motives and goal reengagement, autonomous goal motives foster athletes’ goal reengagement when the situation requires it, whereas controlled goal motives do not contribute to reengaging in other goals. In line with previous findings, these results reflect the positive contribution of autonomous goal motives to future goal striving (Smith and Ntoumanis, 2014). Additionally, they support past literature within the framework of SCM, which has provided evidence about the adaptive role of autonomous goal motives in different contexts such as the school (e.g., Sanjuán and Ávila, 2018) and the sport context (e.g., Healy et al., 2014; Gaudreau and Braaten, 2016; Martínez-González et al., 2021b).

After SEM, moderated mediation analyses were carried out to test the second hypothesis. The results showed that the interaction between perceived empowering and disempowering climates was not significant in predicting athletes’ goal reengagement through goal motives, contrary to expectations. Therefore, these results revealed that, in these mediation models, perceived empowering climate always predicted autonomous goal motives, regardless of the levels of perceived disempowering climate. In contrast, perceived disempowering climate predicted controlled goal motives, regardless of the perceived empowering climate score. Although other studies have found significant interactions between the two climates in their prediction of certain adaptive and maladaptive outcomes (Appleton and Duda, 2016), the current study shows that, in predicting goal reengagement through goal motives, this interaction was not significant. Consequently, perceived disempowering climate did not weaken the positive effect of perceived empowering climate on goal reengagement (through autonomous goal motives or through controlled motives). On the other hand, perceived disempowering climate did not predict goal reengagement, although it had a positive effect on controlled goal motives, which was not buffered by an empowering climate. These findings suggest that the perception of each climate predicts a different type of goal motives in athletes: an empowering climate predicts autonomous motives, whereas a disempowering climate predicts controlled motives. The current results are consistent with past literature that showed the positive relationship between empowering climates and athletes’ more optimal functioning (Duda et al., 2018), in contrast to disempowering climates, which have been related to maladaptive outcomes (Krommidas et al., 2016).

With regard to the study limitations, it is necessary to note that the design was cross-sectional. Further research is needed to examine the relationships of interest longitudinally, in order to understand how goal reengagement processes take place over a sport season and whether changes in motivational climates predict changes in athletes’ goal reengagement through changes in goal motives. Moreover, when motivational climates are examined, some researchers have indicated that the perception of a group or team may differ from the individual perspective (e.g., Papaioannou et al., 2004; Ntoumanis et al., 2012), and so future multilevel analyses could examine team-level perceptions of climate (González et al., 2017; Pineda-Espejel et al., 2017). Finally, it should be kept in mind that the current findings were obtained in university-level athletes, and so future studies could analyze whether these relationships can be generalized to other samples.

In spite of these limitations, the strength of this study is its contribution to the knowledge about how empowering and disempowering climates predict, through goal motives, athletes’ goal reengagement when they have to face unattainable goals. Specifically, it confirmed the importance of empowering climates (independently of the disempowering climate levels) in promoting athletes’ autonomous goal motives, which in turn facilitate athletes’ goal reengagement. Moreover, the results also show that empowering climates do not buffer the effects of disempowering climates on athletes’ controlled goal motives; that is, disempowering climates promote athletes’ controlled goal motives (independently of the empowering climate levels). Although moderation effects were not found in this study, evidence suggests that empowering and disempowering climates may coexist (e.g., Smith et al., 2015, 2017). Taking this into consideration, more research is needed to examine moderation effects and continue to explore how these climates might interact in predicting other important variables in the sport context. In addition to the theoretical contributions, the results of this study are a first step toward further research that includes longitudinal and experimental designs, building on the reported findings. Thus, more evidence will be provided about the importance of not only promoting empowering climates, but also reducing disempowering climates. In this line, athletes may benefit from having a more empowering and less disempowering coach because they will have better well-being (Krommidas et al., 2016) and, as this study shows, better goal adjustment. Thus, educational workshops based on this theoretical framework and addressed to coaches are necessary, such as the empirically evaluated training program Empowering Coaching™ (Duda, 2013). Via different workshops, this program provides coaches with theoretical knowledge and promotes the development of practical skills, in order to generate and maintain more empowering climates while reducing disempowering behaviors (Duda, 2013; Duda and Appleton, 2016; Balaguer et al., 2021). Finally, based on the current study, coaches can be encouraged to take part in these workshops to become more empowering and less disempowering because, in addition to providing benefits for athletes’ well-being, as previous literature has described, they also promote better goal adjustment.

## Data Availability Statement

The datasets presented in this article are not readily available because additional studies are undergoing using the dataset. Requests to access the datasets should be directed to NM-G, natalia.martinez@uv.es.

## Ethics Statement

The studies involving human participants were reviewed and approved by the Human Research Ethics Committee of the University of Valencia. The patients/participants provided their written informed consent to participate in this study.

## Author Contributions

All authors listed have made a substantial, direct, and intellectual contribution to the work, and approved it for publication.

## Conflict of Interest

The authors declare that the research was conducted in the absence of any commercial or financial relationships that could be construed as a potential conflict of interest.

## Publisher’s Note

All claims expressed in this article are solely those of the authors and do not necessarily represent those of their affiliated organizations, or those of the publisher, the editors and the reviewers. Any product that may be evaluated in this article, or claim that may be made by its manufacturer, is not guaranteed or endorsed by the publisher.

## Footnotes

1 Appleton, P. R., Viladrich, C., Balaguer, I., Hall, H., Ommundsen, Y., Papaioannou, A., et al. (under review). *Measurement Invariance of the Empowering and Disempowering Motivational Climate Questionnaire-Coach (EDMCQ-C) in Youth Sport*.
